# Investigation of the effectiveness of hyperthermic intraperitoneal chemotherapy in experimental colorectal peritoneal metastasis model

**DOI:** 10.1515/pp-2023-0002

**Published:** 2023-05-10

**Authors:** Berke Manoğlu, Tuğba Yavuzşen, Safiye Aktaş, Zekiye Altun, Osman Yılmaz, Özde Elif Gökbayrak, Aylin Erol

**Affiliations:** Department of General Surgery, Dokuz Eylul University Faculty of Medicine, Izmir, Türkiye; Department of Clinical Oncology, Dokuz Eylul University Faculty of Medicine, Izmir, Türkiye; Department of Basic Oncology, Dokuz Eylul University Institute of Oncology, Izmir, Türkiye; Department of Laboratory Animal Science, Dokuz Eylul University Faculty of Medicine, Izmir, Türkiye

**Keywords:** colorectal cancer, hyperthermic intraperitoneal chemotherapy (HIPEC), peritoneal metastasis model

## Abstract

**Objectives:**

In our study, we aimed to (1) create a peritoneal metastasis (PM) model in nude mice, administer intraperitoneal chemotherapy using the peritoneal infusion pump we developed in this model, and (2) compare the efficacy of intraperitoneal chemotherapy using various drugs at different temperatures.

**Methods:**

The peritoneal metastasis model was established in nude mice using the CC531 colon carcinoma cell line. Models with peritoneal metastasis (PM) were randomized into four groups of seven animals each: Group 1, control group (n=7); Group 2, normothermic intraperitoneal chemotherapy (NIPEC) with mitomycin C(MMC) (n=7); Group 3, hyperthermic intraperitoneal chemotherapy (HIPEC) with mitomycin C (n=7), and Group 4, NIPEC with 5-fluorouracil (5-FU).

**Results:**

Tumor development was achieved in all animals. While the tumor burden decreased significantly in the treatment Group 3 (p=0.034), no significant difference was found in the other groups. In the PM mouse model, hyperthermic intraperitoneal administration of MMC had the highest tumoricidal effect.

**Conclusions:**

Our PM model provided a good opportunity to examine the efficacy of HIPEC and intraperitoneal infusion pump (IPIP). In future studies, we plan to evaluate efficacies of different drugs in the PM models we have created.

## Introduction

Peritoneal metastasis (PM) clinically represents the malignant progression common in ovarian, colon, and gastric cancer [1]. Ovarian cancer is the leading cause of gynecologic cancer-related death and the fifth cause of cancer death among women. Peritoneal carcinomatosis is diagnosed in the advanced disease stage in 70 % of the patients [2, 3]. Isolated peritoneal transplantation is performed during primary surgery in 8 % of patients with colon cancer, and peritoneal metastases are seen in 25 % of patients with relapse [4, 5]. While an average of one year survival can be achieved with systemic chemotherapy in patients with peritoneal metastases of colorectal cancer (CRC), 5-year survival rates can reach 40–58 % with cytoreductive surgery (CRS) and hyperthermic intraperitoneal chemotherapy(HIPEC) [6]. Therefore, it is recommended that CRS &HIPEC be performed as standard treatment in selected patients [7]. Although they are more successful when compared with systemic chemotherapy, there is not enough scientific evidence about CRS and HIPEC. Although hyperthermia per se has a cytotoxic effect on cancer cells, CRS and HIPEC potentiate each other’s effects with chemotherapy [8]. When compared with systemic chemotherapy, intraperitoneal administration of chemotherapy provides a more intense concentration of chemotherapeutic agents on tumor cells with lower systemic toxicity [9]. Because of all these effects, when HIPEC is applied, 20–50 times more tumoricidal effect occurs compared to systemic chemotherapy [10].

In our study, we planned to create a colorectal PM model in athymic mice by using CC531 (colon adenocarcinoma cell line) and to administer intraperitoneal chemotherapy using the infusion pump we developed in this model. Efficacies of treatment groups were compared by applying different chemotherapeutic agents at different temperatures.

## Materials and methods

Our study was conducted at Dokuz Eylül University Faculty of Medicine Experimental Animals Laboratory (DEUFMEAL) between January and July 2022, with the approval of the University Local Animal Ethics Committee (Protocol No: 03/2018). In the process of establishing the peritoneal metastasis model, 7–8 week-old 28 male athymic nude mice bred by Experimental Animals Laboratory were used. Nude mice being caged in groups of seven under laboratory conditions in air-filtered laminar flow cabinets were monitored. Mice were fed with irradiated food and autoclaved reverse osmosis treated water, and all treatments were carried out under sterile conditions in a laminar flow hood.

### Intraperitoneal tumor cell inoculation

Cancer cells from the CC531 colon adenocarcinoma cell line were harvested during the logarithmic growth stage by incubating them at 37 °C under a humidified 5 % CO₂ atmosphere. Cells were then resuspended in phosphate-buffered saline (PBS) for intraperitoneal injection. By providing the necessary sterilization in the laminar flow hood, suspended cells were given by intraperitoneal (IP) injection using a 16 mm long and 0.45 mm diameter needle. The amount to be injected into all groups was determined as 5 × 10⁶ cells, 0.3 cc, in 200 µL PBS, taking the previous studies as an example [8]. We detected development of distension and palpable nodular lesions due to the formation of intraabdominal ascites between the 7th and 10th days in the subjects who were checked daily by inspection and palpation starting from the 5th day. After the presence of tumor was detected (day 10), the subjects were divided into four groups: Group-1 (G-1), control group, (0.9 % NaCl); Group-2 (G-2), normothermic chemotherapy with mitomycin C (MMC); Group-3 (G-3), hyperthermic chemotherapy with MMC; Group-4 (G-4), normothermic chemotherapy with 5-fluorouracil (5-FU) groups (Table 1).

**Table 1:** Groups and intraperitoneal treatment procedures.

Groups	Intraperitoneal treatment procedure
G 1 (control)	Normothermic(37°) 0.9 % NaCl
G 2 (normothermic MMC)	Normothermic(37°) MMC
G 3 (hyperthermic MMC)	Hiyperthermic(41°) MMC
G 4 (normothermic 5 FU)	Normothermic(37°) 5 FU

MMC, mitomycin C; 5-FU, 5-fluorouracil.

### Surgical intervention and HIPEC procedure

**Figure 1:**
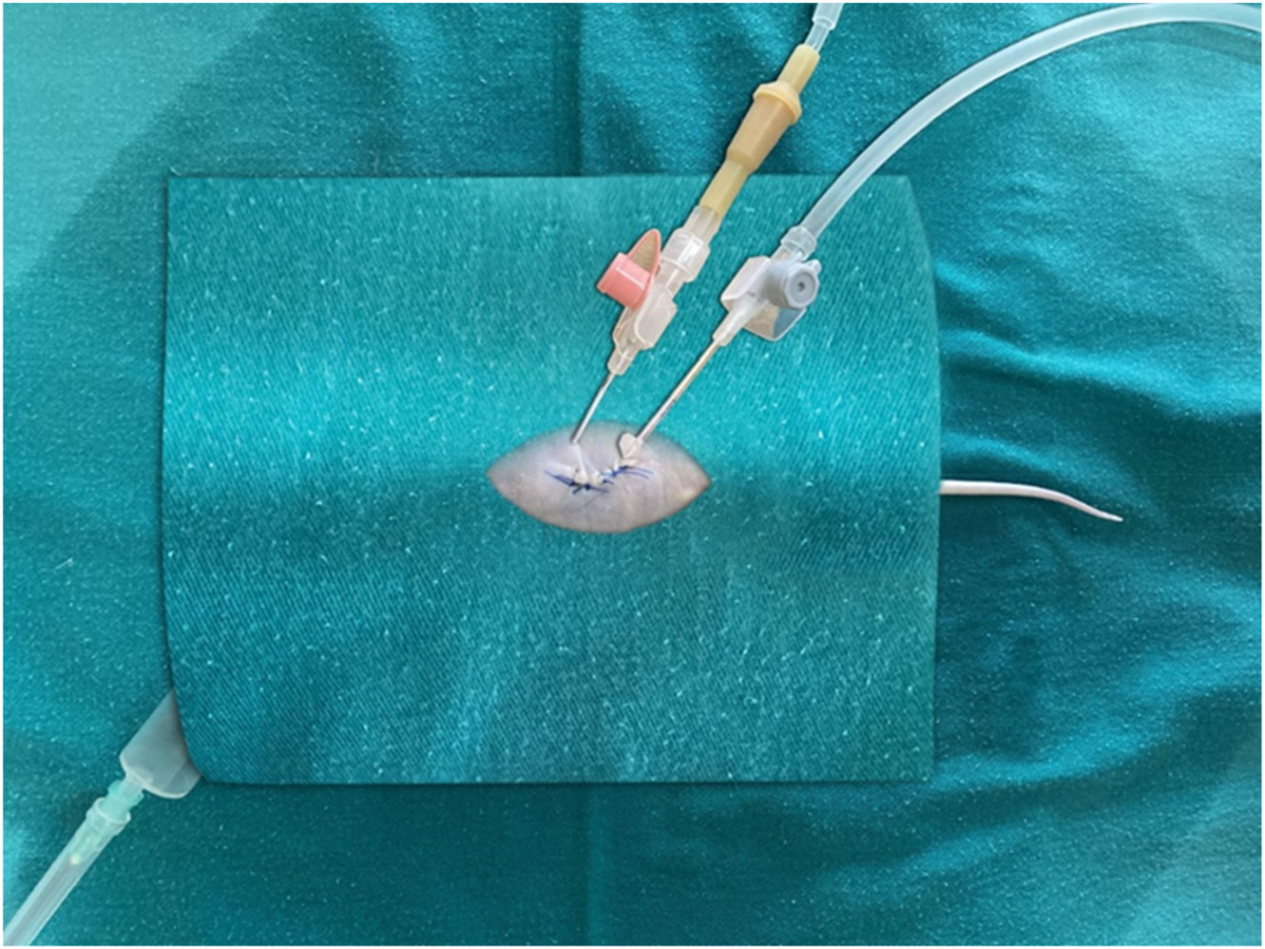
Preparation of the model before the application of intraperitoneal chemotherapy, placement of the catheters.

**Figure 2:**
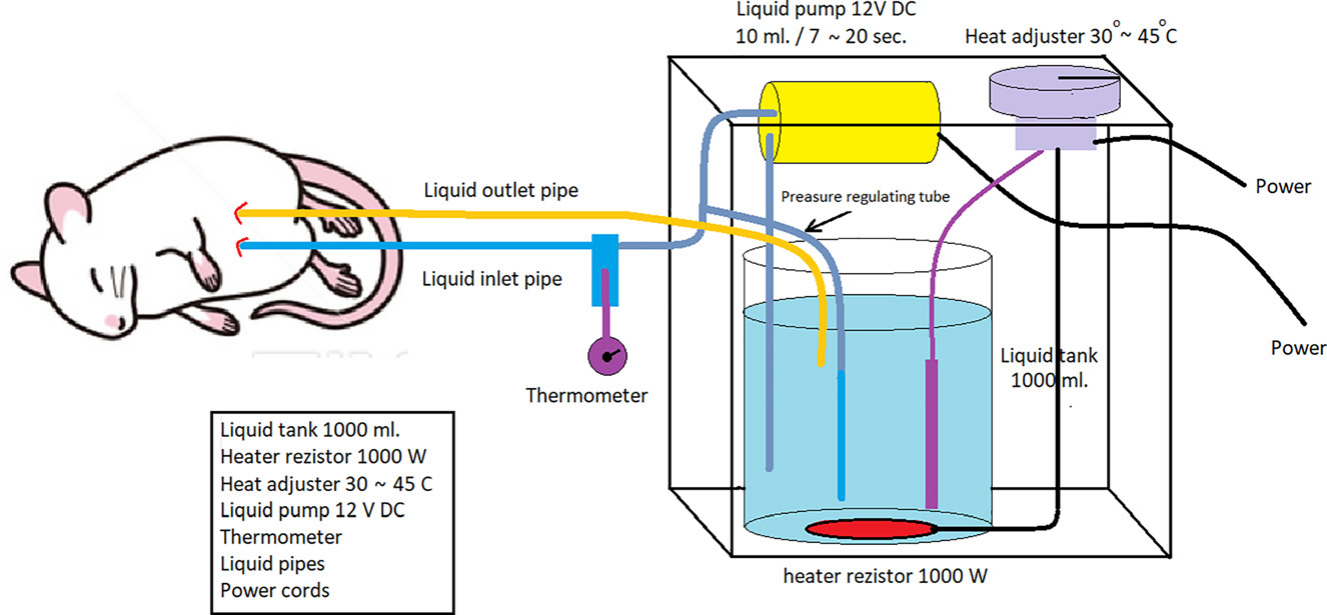
Intraperitoneal infusion pump system working diagram.

Athymic nude mice were weighed before administration of anesthesia. Their mean weight was 34 ± 2 g. Diethyl ether inhalation anesthesia was applied. After anesthesia; the abdominal skin was cleaned with povidone-iodine. Necessary sterilization conditions were provided by covering the mouse with sterile covers. A midline abdominal incision of approximately 1 cm was made and the abdomen was entered. Peritoneal metastasis was found. After the inlet and outlet catheters of the intraperitoneal infusion pump (IPIP) were placed lateral to the abdomen, the midline incision was closed primarily with 4/0 prolene sutures (Figure 1). Then, different chemotherapeutic agents were infused under normothermic (37 °C) (5- fluorouracil 400 mg/m^2^, mitomycin C 20 mg/m^2^), and hyperthermic (41 °C) (mitomycin C 20 mg/m^2^) conditions for 45 min to the previously determined groups. This process was developed by us as a prototype and was carried out with IPIP (Figure 2). Intraoperatively, the required tissue temperature was reached within 4–6 min in the HIPEC group. Steady temperatures were then maintained for an additional 45 min with an average of 40.5 ± 0.5 °C, and approximately 5 mL of the solution was required to fill the abdomen. Using the thermostat of the device, the temperature of the fluid given and instilled into the abdomen was controlled and the temperature was kept constant. Chemotherapeutic agents were given as MMC (20 mg/m^2^), and 5-FU (400 mg/m^2^) prepared in 30 cc 0.9 % NaCl. This process was done in a laboratory environment, taking safety precautions. Body surfaces of mice were calculated in square meters (m^2^) using the formula: (A(m^2^)=K × W⅔/100). Intraperitoneal perfusion was maintained for 45 min. After the procedure was completed, the perfusion cannulas were taken out of the abdomen. The liquid boiler of the system was sterilized after each operation. All infusion tubing and cannulas were changed after each procedure. Sterilization was provided under optimal conditions for each mouse.

### Intraperitoneal infusion pump (IPIP)

**Figure 3:**
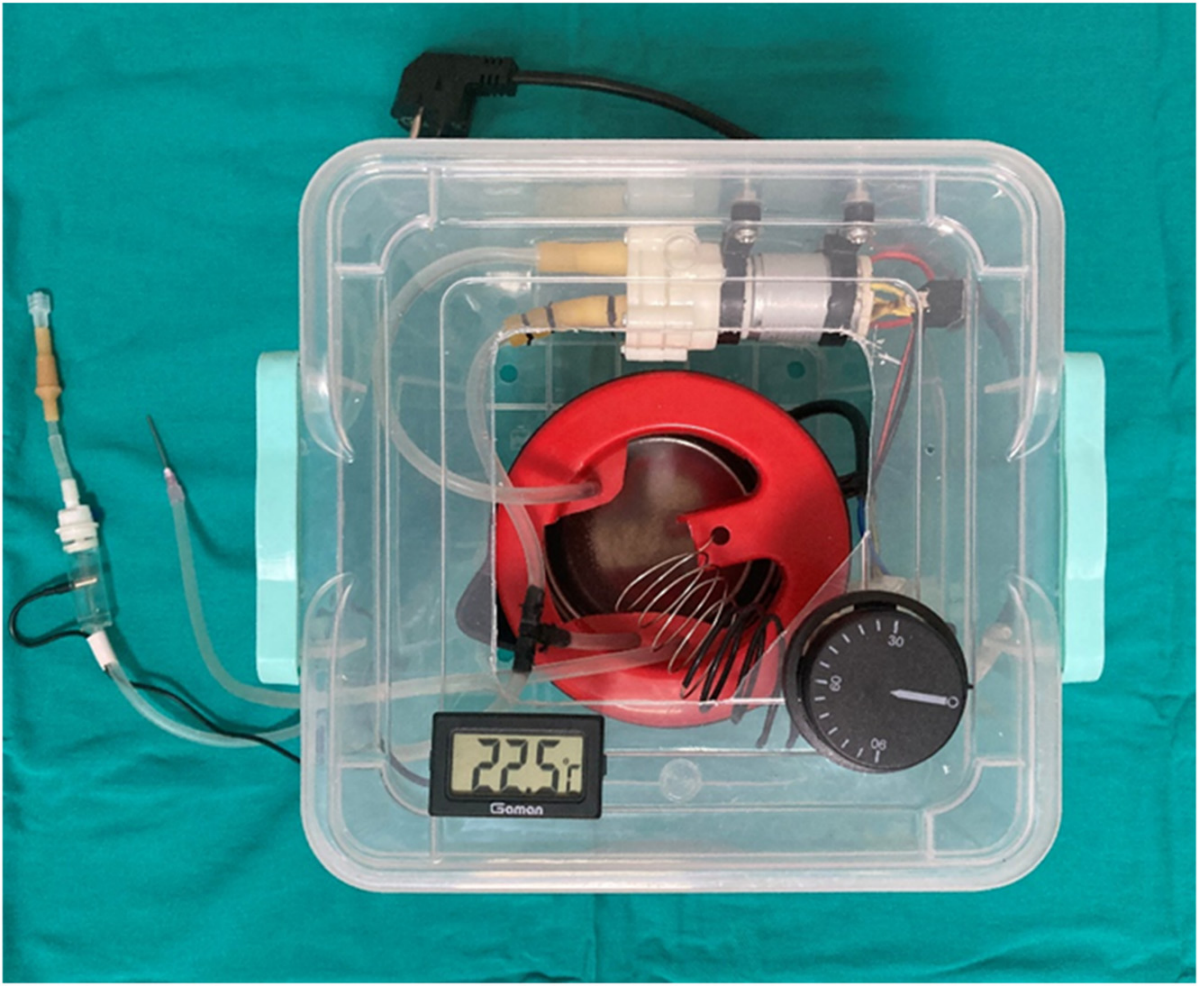
Intraperitoneal infusion pump (IPIP).

It consists of intraperitoneal infusion pump (IPIP), 1,000 mL liquid tank, liquid pump, thermostat that can control the range of temperature between 30 and 45 °C, heater resistance (1000 W), liquid temperature control probe, liquid flow and collection cannulas, liquid pressure adjustment cannula designed and developed by us (Figure 3). The temperature of the fluid coming from the fluid outlet cannula and the inside of the abdomen passes through the second control cannula and a constant temperature is provided. The flow rate of the liquid pump can be adjusted. Intraperitoneal chemotherapy can be applied to colorectal PM models created in different types with the IPIP system we have developed.

### Follow-up, sacrification and evaluation of subjects

**Figure 4:**
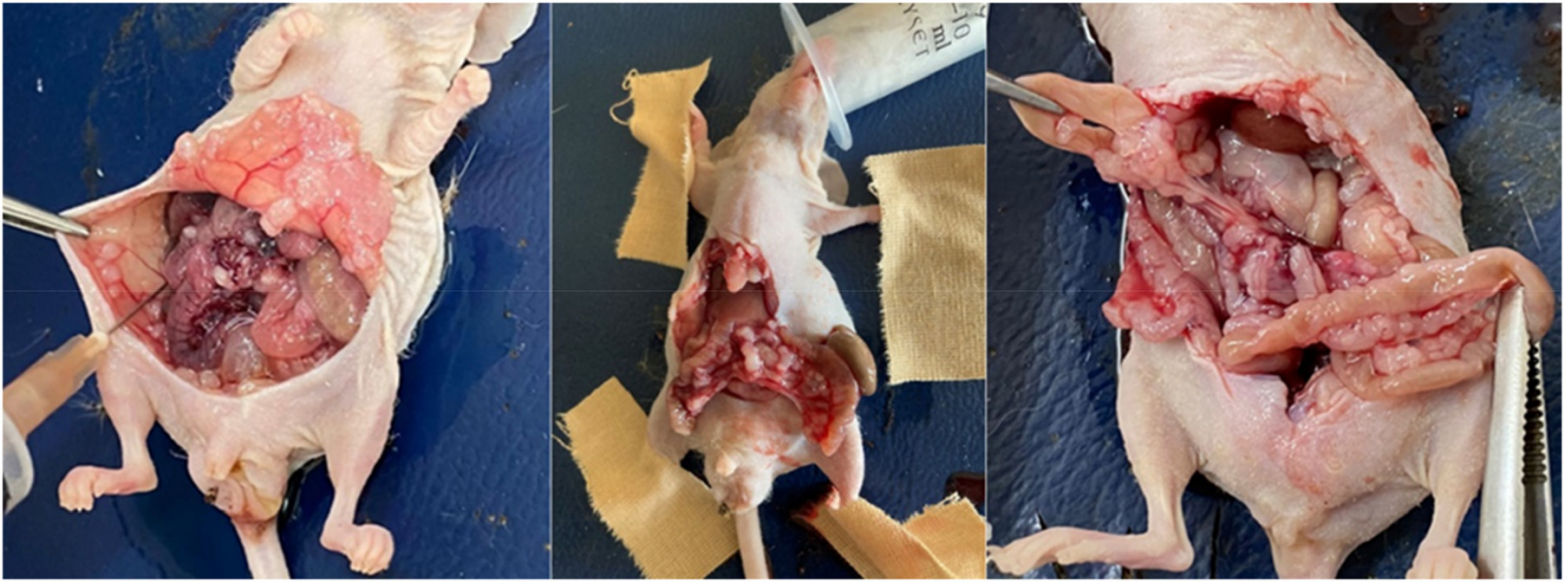
Determination of peritoneal carcinomatosis index in animals, acid amount measurement.

Subjects undergoing daily follow-ups were evaluated by performing laparotomy 5 days after intraperitoneal infusion of chemotherapeutic agents. Peritoneal cancer index (PCI) was determined and scoring was done. Scoring was done considering the involved organ and tumor diameter, and evaluated out of 8 points as follows: small bowel and/or mesenteric involvement: 1 point; peritoneal involvement: 1 point; diaphragmatic involvement: 1 point, ascites (+): 1 point; involvement of other organs: 1 point. Tumor diameters were measured and scored as follows: 0: no tumor growth; 1 point: nodule diameter ≤2 mm; 2 points: nodule diameter 2–5 mm or >5 tumor nodules; 3 points: nodule diameter ≥5 mm or >10 tumor nodules (Figure 4). Ascitic fluid was aspirated, and its quantification was carried out. Small intestine, peritoneum, intra-abdominal fluid and blood samples were taken and the subjects were sacrificed. Tissue and intra-abdominal fluid samples were then evaluated histopathologically and biochemically. Tissue samples were fixed in 10 % formaldehyde, cassetted, and embedded in a paraffin block after tissue follow-up. Frozen sections of 5 µm thickness were obtained from the optimum section surface. Sections were then stained with hematoxylin and eosin (H&E) and examined under Olympus X50 light microscope. Tissues were evaluated for the presence of tumor, tumoral pattern, differentiation, apoptosis, mitosis and necrosis. Evaluation was made by calculating the total number of mitoses in 10 different tumor areas by magnifying the field of vision 400 times under a 40X objective of a light microscope. The number of apoptosis was calculated by evaluating 5,000 cells and determining its percentage in 1,000 cells. Tissue samples were evaluated for tumor necrosis. Supernatants remaining after centrifugation of intra-abdominal fluid samples of the mice were studied using lysyl oxidase-like protein 1(LOXL1) and TWIST Transcription factor (TWIST) mouse compatible ELISA kits. Vascular endothelial growth factor (VEGF) levels were studied by diluting the samples in fluids, taking into account the mouse-compatible ELISA kit application steps. According to the absorbance values obtained from the standards, standard graphs of each test were created. Concentrations were expressed by calculating the absorbances obtained from the samples. The measuring range of the LOXL1 ELISA kit was 78–5,000 pg/mL and the measurement sensitivity of the test kit was 29 pg/mL. The measuring range of the kit for the TWIST test was 0.156–10 ng/mL, and the measurement sensitivity of the test kit was 0.056 ng/mL. The measuring range of the kit for the VEGF test was 15–1,000 pg/mL, and the measurement sensitivity of the test kit was 9.375 pg/mL.

### Statistical analysis

Before the study, the number of subjects was determined by power analysis. The maximum number of animals allowed by the animal experimentation ethics committee was used to obtain statistically significant results. Statistical analysis was performed using IBM SPSS 24.0 statistics. The significance of differences was assessed by the Kruskal-Wallis-test. Continuous variables were compared by independent samples t-test. Descriptive statistics were presented in median (25–75th percentile) format. Fisher exact chi-square test and T-test were used in the analysis of qualitative data, and descriptive statistics were shown in the form of frequency. p-Values <0.05 were defined as statistically significant.

## Results

There was no mortality in the postoperative period in the mice that received intraperitoneal chemotherapy. Mild side effects (anorexia and lethargy) were observed in four animals in the group given hyperthermic chemotherapy which disappeared within two days. Although minimal dehiscence was observed in the incision line in nine animals, no infection or wound dehiscence, which would cause mortality, was observed until the sacrification process.

### Macroscopic findings

When the peritoneal cancer index (PCI) was compared between the groups, the group given hyperthermic intraperitoneal MMC (Group 3) had the lowest mean and statistically significant PCI (p=0.034) value [(6.42 ± 1.71)]. When tumor diameters and amount of ascites were compared between groups, Group 3 again had the lowest, and statistically significant PCI values (5.28 ± 2.28 mm vs. 3 ± 2.16 mL, and p=0.011 vs. p=0.046, respectively). However, in our study, no significant difference was found between the groups in terms of these parameters, except for Group 3 (Table 2).

**Table 2:** Group 3 mean PCI, tumor diameter and amount of ascites were significantly less than the other groups (Kruskal-Wallis test, t-test).

Mean(std±)	G-1	G-2	G-3	G-4	p-Value
PCI	6.71 (±1.38)	6.42 (±1.71)	4.42 (±0.53)	6.42 (±1.39)	0.034
Tumor diameter, mm	5.85 (±3.28)	5.28 (±2.28)	2.42 (±0.53)	5.42 (±1.90)	0.011
Ascite, mL	4 (±1.93)	3 (±2.16)	1.28 (±1.77)	4.21 (±0.63)	0.046

PCI, peritoneal cancer index; std, standard deviation.

### Microscopic findings

**Figure 5:**
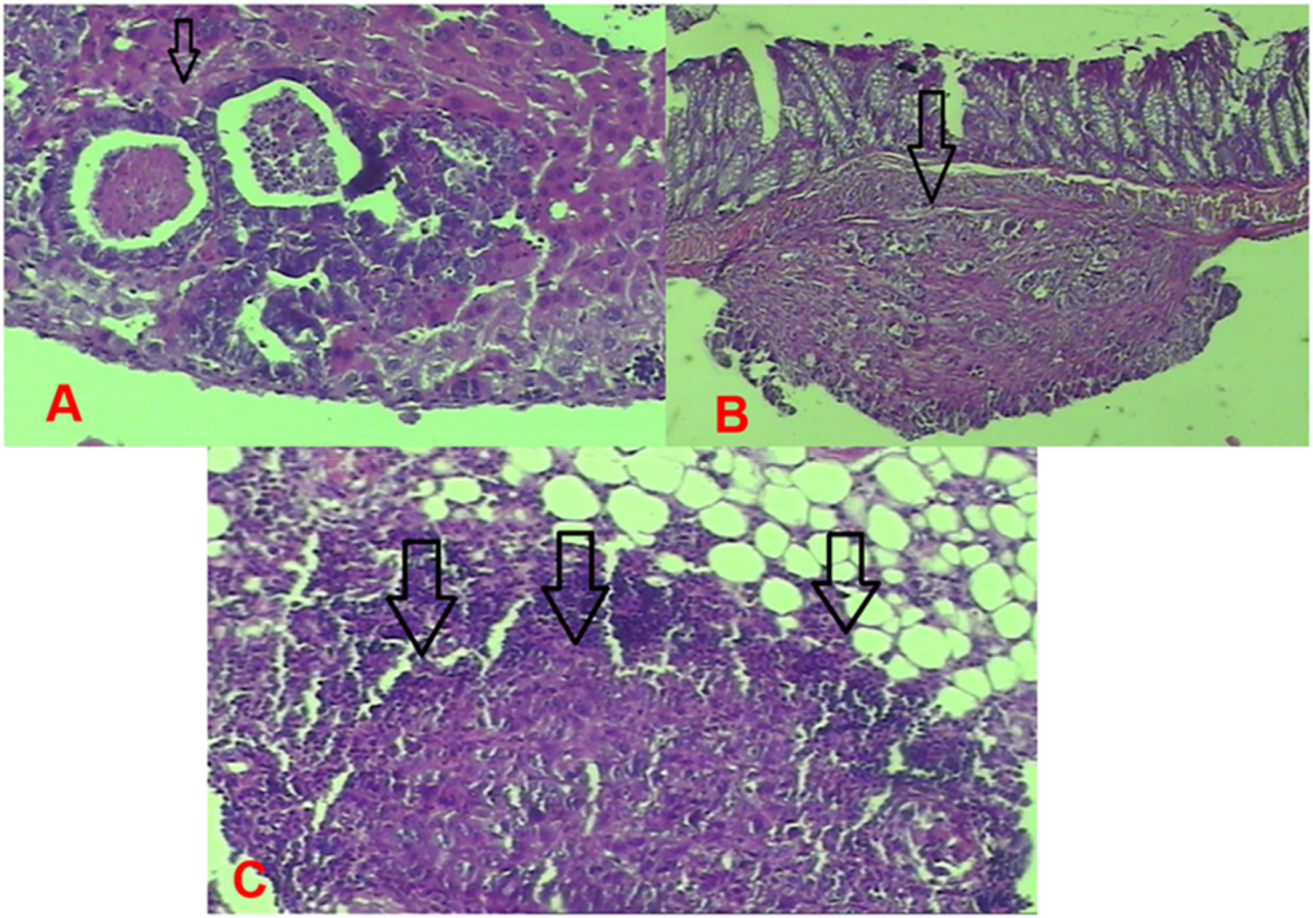
Group 1 (control) tumor images. (A) Nodular tumor implanted in the liver. (B) Tumor implantation in the intestinal wall. (C) Tumor infiltrating the peritoneal adipose tissue.

**Figure 6:**
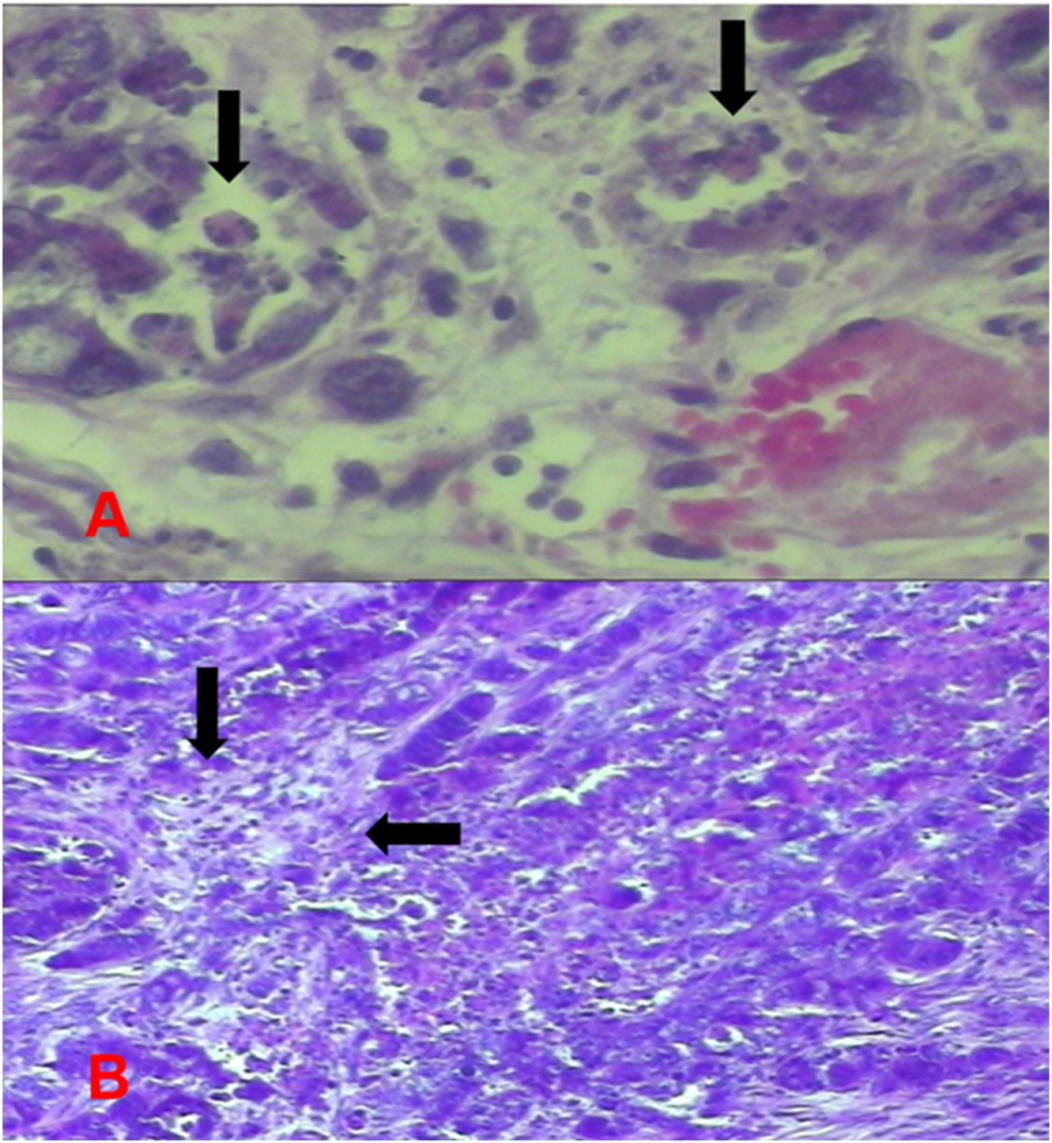
Group 3 (hyperthermic MMC) tumor images. (A) Increased apoptosis in tumor cells. (B) Areas of tumor necrosis.

When the tissue samples obtained from the intestinal system, peritoneum, and liver after sacrification were evaluated under microscope, tumor cell infiltration was observed in all tissues. The tumor was found to be nodular and undifferentiated. When the groups were compared in terms of number of mitotic, and apoptotic cells and tumor necrosis, statistically significant intergroup differences were found (p<0.01). In the hyperthermic intraperitoneal MMC (Group 3) group, the number of apoptotic cells and areas of tumor necrosis were found to be statistically significantly higher than the other groups (p<0.001, p=0.009). The number of mitosis in the normothermic intraperitoneal MMC (Group 2) group was statistically significantly lower than the control group (G 1) and normothermic 5 FU group (G 4) (p<0.001). No significant difference was found between the hyperthermic MMC group (G 3) and the normothermic MMC group (G 2) in terms of the number of mitotic cells (Table 3, Figures 5 and 6).

**Table 3:** The mean of tumor tissues in the groups; mitosis counts, apoptosis counts and tumor necrosis rates (Kruskal-Wallis test, t-test, p<0.001, p=0.009).

	Mitosis count (40×)	Apoptosis count/1,000 cell	Tumor necrosis (+) subject ratio in groups, %
G 1 (control)	12.714(std 1.79)	5.42(std 2.29)	14.2
G 2 (normothermic MMC)	4.285(std 1.97)	71.42(std 37.16)	28.5
G 3 (hyperthermic MMC)	5.857(std1.34)	131.42(std 48.79)	100
G 4 (normothermic 5 FU)	7.285(std 2.49)	72.14(std 29.98)	28.5
p	<0.001	<0.001	0.009

MMC, mitomycin C; 5-FU, 5-fluorouracil; std, standard deviation.

### Biochemical findings

The mean values of VEGF, LOX1 and TWIST in the intra-abdominal ascites fluid were found to be statistically significantly lower in G3 compared to the other groups (VEGF; 17.960 ± 24.728 pg/mL, LOX1; 225.000 ± 308.801 pg/mL, TWIST; 0.254 ± 0.407 ng/mL [p=0.011, 0.042, and 0.044, respectively]). No significant difference was found between the other groups in terms of these parameters (Table 4).

**Table 4:** VEGF, LOX1 ve TWIST values in intra-abdominal fluid (Kruskal-Wallis test, t-test, p=0.011, 0.042, 0.044).

	VEGF	LOX1	TWIST
G 1 (control)	274.625 (std±192.498)	552.142 (std±326.622)	1.416 (std±1.164)
G 2 (normothermic MMC)	137.687 (std±113.335)	382.857 (std±303.422)	1.565 (std±1.258)
G 3 (hyperthermic MMC)	17.960 (std±24.728)	225.000 (std±308.801)	0.254 (std±0.407)
G 4 (normothermic 5 FU)	103.640 (std±62.946)	750.714 (std±362.254)	2.176 (std±1.498)
p	0.011	0.042	0.044

VEGF, vascular endothelial cell growth factor; LOX1, lysyl oxidase like protein 1; TWIST, twist transcription factor; std, standard deviation.

## Discussion

In our study, we aimed to create a peritoneal metastasis model in athymic mice and to compare the efficacy of different temperatures and drugs by giving intraperitoneal chemotherapy with the peritoneal infusion pump we developed in this model.

HIPEC with mitomycin C has been applied following cytoreductive surgery for various peritoneal surface malignancies. Spratt et al. first performed HIPEC in a patient with pseudomyxoma peritonei [11]. A significant survival benefit has been shown for HIPEC when compared to systemic chemotherapy alone [12, 13]. The complete cytoreductive surgery is the most important prognostic factor. Incomplete cytoreduction results in limited survival [14, 15].

The drugs used in the HIPEC procedure have a limited depth of penetration. For this reason, HIPEC is applied in patients whose macroscopic tumor burden was eliminated or minimal residual tumor remained following radical cytoreductive surgery [16]. Therefore, tumor cells can be implanted into an intraperitoneal fat pad to simulate cytoreductive surgery as described by Veenhuizen et al. [17]. Using this technique, the spread of tumor implants is limited. This simulates an abdomen that has undergone cytoreductive surgery and has a reduced tumor burden. In our study, we provided widespread implantation of tumor cells by injecting tumor cells into the intraperitoneal cavity. Diffuse peritoneal implants formed in all subjects within 7–10 days. In many studies, and tumors are produced by intraperitoneal injection [18, 19]. The widespread creation of peritoneal implants made it easier for us to determine the macroscopic PCI score. Apart from this, we think that this approach enables us to better detect the differences in efficacies of different drugs administered to the groups. In some studies, the tumor formation rate after intraperitoneal tumor transplantation was reported as 80 % [20, 21], while tumor formation rate of 100 % was reported in a study where tumor cells were implanted in an intraperitoneal fat pad [17]. In our study, tumor formation was observed at a rate of 100 % after intraperitoneal inoculation.

We have seen that with the IPIP system we developed, HIPEC can be performed effectively in the athymic mouse PM model. There was no loss of subjects during and after perfusion. Animals were observed for 5 days after administration of intraperitoneal chemotherapy. No serious complications were observed. Mild side effects (anorexia and lethargy) were observed in four animals in the group given only hyperthermic chemotherapy. All these side effects disappeared within two days. Late-term effects, morbidities, and effects of sacrification on the 5th day could not be fully evaluated. Basically, HIPEC is a proven procedure with cytoreductive surgery. However, in our study, only HIPEC was applied since it was not appropriate to perform cytoreductive surgery on the model. In this case, the administered chemotherapeutic drugs demonstrated limited effectiveness.

It is difficult to achieve homogeneous distribution of temperature, and cytotoxic drugs, but it is crucial for ensuring the tumoricidal efficacy of this procedure. For this reason, an open abdomen approach can be chosen to ensure homogeneous temperature and drug distribution [22]. The biggest disadvantage of this method is exposure to cytotoxic drugs. In our study, after perfusion catheters were placed, the abdominal wall was closed and then peritoneal infusion was started. Optimal intraperitoneal circulation was ensured by continuous temperature control and adjustment of the infusion rate in the fluid outflow and inflow catheters, and the desired temperature and homogeneous drug distribution were maintained. The closed abdomen facilitated the control and maintenance of the same drug temperature. After the treatment period was completed, the cannulas were withdrawn and the procedure was terminated.

In cases of peritoneal metastasis of colorectal cancer, MMC is the most common chemotherapeutic agent used for HIPEC. Another intraperitoneal chemotherapy modality is early postoperative intraperitoneal chemotherapy (EPIC). The 5-fluorouracil is mainly used for EPIC [23, 24], and it is not used in hyperthermic applications. In our study, MMC was applied in normothermic (Group 2) and hyperthermic (Group 3) applications. In Group 4, 5-FU was applied normothermically. The drug efficacy in these three groups was compared. The highest tumoricidal activity was observed in Group 3. In animal models, experimental studies have shown that MMC is quite potent and inhibits tumor growth at the maximum tolerated IP dose of 20 mg/m^2^ [25]. The therapeutic concentrations of MMC (20 mg/m^2^) and 5-FU (400 mg/m^2^) we applied in our study were determined in accordance with the studies in the literature [24], [25], [26], [27], [28].

The results of our study confirm the clinical data collected in animal models of peritoneal metastasis where intraperitoneal chemotherapy was used [29]. The group in which hyperthermic MMC was applied (G 3) demonstrated the highest tumoricidal activity. No statistically significant difference was found between the other groups in terms of tumoricidal activity. In microscopic examinations; in the hyperthermic MMC-applied group, higher rates of apoptosis and tumor necrosis but lower number of mitoses were observed in the tumor cells detected in the sections of tissue samples which demonstrated higher tumoricidal efficiency of the hyperthermic treatment relative to the other groups. The rates of apoptosis and tumor necrosis were higher but the number of mitoses was lower in the other treatment groups compared to the control group. As a result of biochemical evaluations, VEGF, LOX1, and TWIST values in ascitic fluid were found to be significantly lower in the hyperthermic MMC group compared to the other groups. When we evaluate all the findings, macroscopic, microscopic and biochemical examination results jointly have shown that the strongest tumoricidal activity was achieved in the hyperthermic MMC group.

Limitations of this study can be stated as small number of mice included in the nude mouse peritoneal carcinomatosis model, and very difficult application of chemotherapy procedure. The mice were followed up and sacrificed until the 5th postoperative day. Therefore, the long-term efficacy of the drugs and the late-term postoperative complications could not be evaluated. Due to the limited number of studies in this area, it was not possible to foresee the difficulties that may be encountered. Before this experiment, preliminary experimental studies were carried out in order to establish peritoneal metastasis and to gain experience in IPIP procedure.

## Conclusions

Hyperthermic intraperitoneal chemotherapy procedure can be applied in the created peritoneal metastasis model and the results can be evaluated qualitatively and quantitatively. The effect of HIPEC can be evaluated macroscopically. Postoperative observations for 5 days can be made in nude mice with acceptable morbidity rates. Intraperitoneal chemotherapy was applied effectively with the peritoneal infusion pump we have developed. In future studies, IPIP can be used reliably on animal models. We compared tumoricidal efficacies of the drugs used; found that statistically significantly higher tumoricidal efficacy had been achieved in the hyperthermic MMC group. In subsequent studies, we have planned to test different drug activities at different temperatures with this model we created and the IPIP system we developed. We also aim to further increase the efficiency of the HIPEC procedure and further reduce its side effects by conducting experimental *in vitro* and *in vivo* studies in the future.
